# Geoelectrical resistivity and geochemistry monitoring of landfill leachates due to the seasonal variations and the implications on groundwater systems and public health

**DOI:** 10.1038/s41598-024-77727-6

**Published:** 2024-11-03

**Authors:** Joseph Omeiza Alao, Oche Joseph Otorkpa, Fahad Abubakar, Daniel Eshimiakhe, Abubakar Aliyu, Momohjimoh Abdulsalami, Danga Onimisi Abdulmalik

**Affiliations:** 1 Department of Physics, Air Force Institute of Technology, Kaduna, Nigeria; 2 Department of Public Health, School of Public Health, Texila American University, Georgetown, Guyana; 3 Department of Geosciences, Confluence University of Science and Technology, Osara, Okene, Nigeria; 4 Department of Physics, Ahmadu Bello University, Zaria, Nigeria; 5 Department of Physics, University of Maiduguri, Maiduguri, Nigeria

**Keywords:** Geoelectrical data, Water analysis, Leachate plumes, Seasonal variation, Heavy metals, Groundwater, Public health, Environmental sciences, Solid Earth sciences, Physics

## Abstract

Understanding the seasonal variations in the landfill leachate plumes (LLPs) properties and complex connections between concentrations of leachate variability, and its environment is essential for environmental and public health management. This study explores the combined electrical resistivity (ER) data and physiochemical water analysis (PWA) coupled with the excavations to monitor the landfill physiochemical properties (LPPs) due to seasonal variations and their implications on environmental vital organs and public health. The variations in ER and LLP distributions across the overburdened top layer due to seasonal changes were examined. The low ER contrasts were encountered within the ranges of 1.5 Ωm – 19.0 Ωm which was mapped as LLP accumulated zones within the landfill, while high ER values varied between 15 Ωm – 260 Ωm off-the landfill extending beyond 15 m. The results of the PWA indicate high concentrations of heavy metals (HMs) such as iron (Fe), lead (Pb), zinc (Zn), and cadmium (Cd) decreasing with wet seasons and increasing with dry seasons. The overall high concentration of HMs in the LLPs was indeed varied between 9.81 ± 2.15–19.07 ± 3.68, while the electrical conductivity (EC) significantly increased from 17.99 ± 1.92 mg/L to 24.87 ± 3.31 mg/L towards the wet season. The increment and decrement encountered in the LPPs are due to seasonal variation and dilution. The order of decrement in the HMs in the LLPs treads as follows EC > Fe > Zn > Pb > Cd in values, respectively. The near-surface EC aligned well with the ER results and boundaries of the waste disposal site, which was verified by the soil excavations. In addition, the ER method was extended beyond the landfill for adequate monitoring, identifying the subsurface layers, conductive shallow zones mapped as the zones of LLP accumulation, resistive deep and shallow zones mapped as the consolidated lateritic topsoil and crystalline basement rocks in some cases, and a dipping conductive lineament zones identified as fracture zones just before the crystalline basement. In conclusion, the ER technique reveals the vertical and horizontal extents of the LLP escapade, the PWA expressed the concentrations of HMs in the LLPs, heightening the implications on the environmental and human health. Finally, the combined techniques deployed for monitoring the physiochemical properties of LLPs due to seasonal variation and the impacts on the integrity of groundwater quality systems and public health inform sustainable waste management practices, which contributes significantly to the protection of groundwater resources and the development of effective strategies to safeguard groundwater systems and public health for present and future generations.

**Supplementary Information:**

The online version contains supplementary material available at 10.1038/s41598-024-77727-6.

## Introduction

Landfills remain an essential component of modern waste management systems, which serve as repositories for different kinds of municipal solid waste (MSW) generated by anthropogenic activities. Landfills have been identified as one of the major environmental concerns that threaten groundwater systems, soil, and air conditions^1^, which inversely affect public health. Landfills remain the major source of leachate plume generation^2,3^, which usually occurs in the form of liquid, especially when rainwater infiltrates the solid waste within the landfill and picks up contaminants, creating a potential risk to groundwater quality^4,5^. Monitoring landfill leachate is crucial for assessing its impact on the surrounding environment, particularly the groundwater system^6,7^. The combined geo-electrical resistivity and physiochemical water analysis monitoring have emerged as one of the valuable geophysical tools for studying the movement and distribution of leachate within landfills^8,9^, providing insights into how seasonal variations influence leachate behaviour and its implications on groundwater quality. Several studies have shown that landfill leachate plumes (LLPs) are responsible for a large percentage of heavy metals in water, impacting both environmental organs and public health negatively^10,11^.

Understanding the behaviour of leachate within landfills is essential for assessing the risks posed to groundwater resources, which are vital sources of drinking water for human populations. Contaminants present in leachate can infiltrate the groundwater system^12,13^, leading to potential environmental degradation and health hazards^11,14,15^. By monitoring the geo-electrical resistivity of landfill leachate, researchers can track the spatial and temporal variations in leachate distribution, identifying areas of high contamination risk and informing effective remediation strategies^16,17^. Seasonal variations play a critical role in shaping the dynamics of leachate generation and movement within landfills^18,19^. During wet seasons, increased precipitation can enhance leachate production by accelerating the percolation of water through the waste materials, resulting in higher leachate volumes and concentrations of contaminants. In contrast, dry seasons may lead to reduced leachate generation as water infiltration decreases, impacting the overall quality and quantity of leachate within the landfill^20,21^. Geo-electrical resistivity monitoring offers a non-invasive and cost-effective approach to studying landfills’ subsurface characteristics and assessing leachate’s behaviour over time.

By integrating geo-electrical resistivity monitoring with hydrogeological modelling and groundwater sampling, researchers can gain a holistic view of the processes governing leachate migration and its impacts on groundwater quality^22,23^. Applying geophysical techniques to study the movement and distribution of leachate within landfills has enhanced the understanding of how seasonal variations influence leachate behaviour and its implications on groundwater quality^24–26^. This research is essential for informing sustainable waste management practices and safeguarding groundwater resources for future generations. The application of electrical resistivity imaging (ERI) to delineate the subsurface distribution of leachate within landfills and monitor changes over time has proven successful across the globe^27,28^. This is because ERI measurements of landfill subsurface can identify the zones of high and low permeability, pathways of leachate migration, and track changes in leachate composition can be identified, which is crucial for understanding the potential pathways through which contaminants can migrate from landfills to groundwater systems^10^. According to previous studies, the concentrations of HMs are usually distributed across the uppermost stratum, which had been previously discovered by ERT studies. As a result^15,17^, the ERT data acquisition is limited to the uppermost overburdened topsoil layer using 3.0 m electrode spacing. This can also provide insight into the regional groundwater vulnerability for critical investigation^29,30^. In conclusion, this study investigates landfill subsurface geo-electrical resistivity in three different seasons to monitor the influence of seasonal variation of LLPs and its potential impacts on the groundwater system.

### Side description

**Fig. 1:**
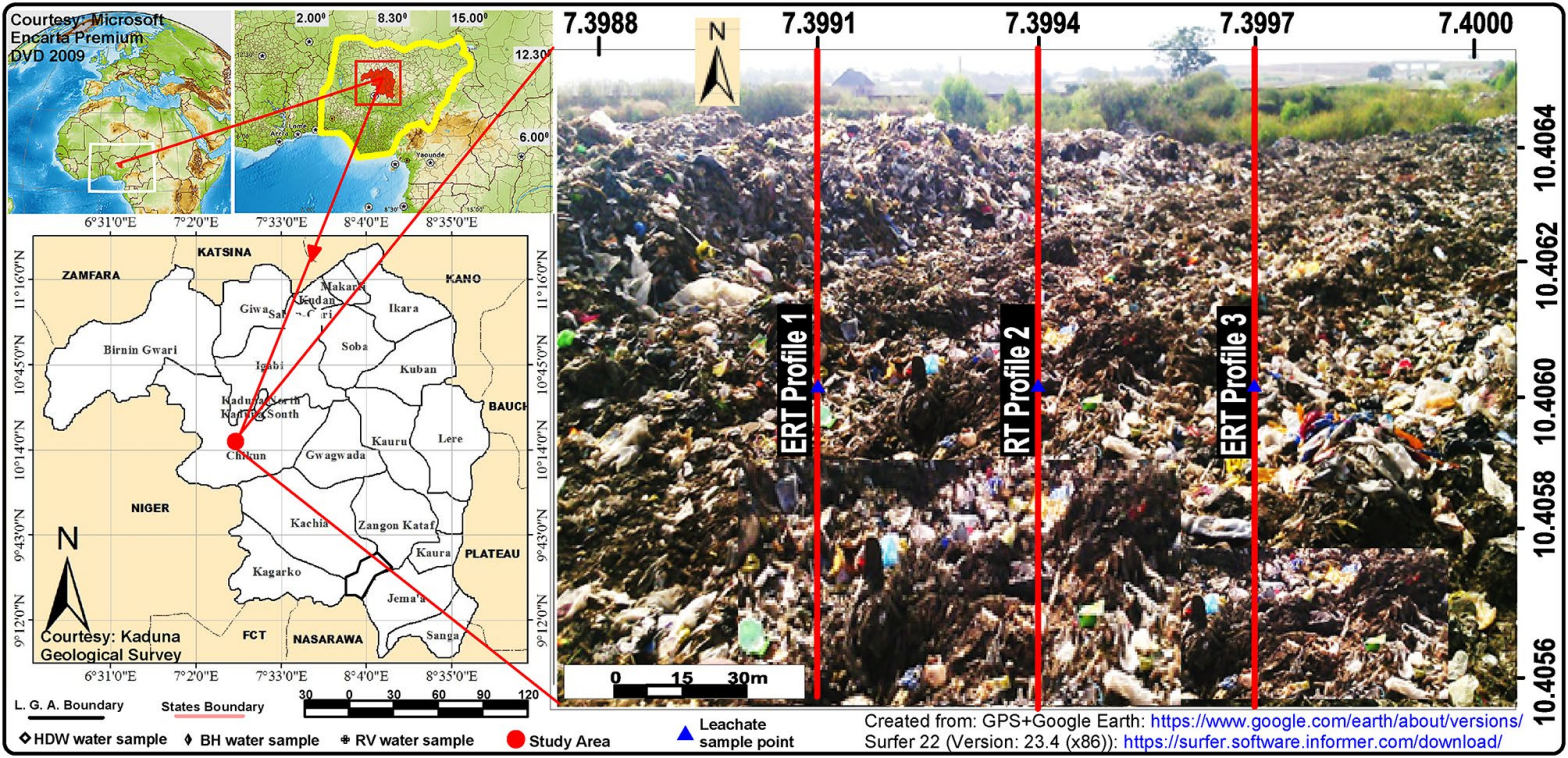
The Map of the Study Area Showing the Landfill and ERT profiles.

The investigation landfill is situated outskirts of Kaduna city limits in the northern region of Nigeria, It is a mini-landfill located in Goni-Gora, Kaduna, on the coordinates of latitude 10°24’22.32” N and longitude 7°20’20.76"E (Fig. 1). Figure 1 was prepared from Google Earth using geographical coordinates acquired by a GPS and a picture of the landfill, which was subsequently prepared using different drawing and plotting tools such as Surfer-22 (Version: 23.4 (x86))^31,32^. According to the previous study, the landfill has been in operation for the past twenty-two years^1^. The landfill extends across a total area of 12,000 square meters, with an average elevation above sea level of 626 m (Kakuri NE sheet 144. 1:50,000). The landfill is estimated with a capacity of about 36,400 $\:{\text{m}}^{3}$. It is composed of mixed garbage with an average height of 3.9 m and a maximum elevation differential of 5 m between the top of the solid waste and the surrounding land. The site geology belongs to the traditional Precambrian Crystalline Basement complex and the Cretaceous-Tertiary sedimentary rocks comprise the two primary lithological units that make up Nigeria’s general geology^33^. The spread of complicated basement rocks characterizes Kaduna’s regional geology. However, over time, these transparent basement rocks have experienced a variety of adversities and deformations of differing degrees, which has caused the rocks to fold and fracture^34^.

This deposit is unique because of its undulations in addition to the bed fracturing and jointing. Seldom near the surface, mixed laterites, sandstones, quartzites, and silty soil occasionally cover the rocks in the district^35^. Throughout the years, the laterites in the area have persisted into lateritic nodules as opposed to ammophilous clays and silty soil^34,36^. According to studies, profound chemical weathering and river erosion have had a significant impact on the bioclimatic character of the Kaduna environment^1,37^, resulting in high undulating plains. As a result, the region’s aquifer unit is made up of clayey or weathered sand and a fractured basement^33^, which usually occurs within a depth of 5.0 m and above^30,38^. The weathered/fractured layer, which constitutes the major regional groundwater units contains overburdened topsoils of about 10.0 m thickness on average were suspected characteristic of relatively high electrical resistivity values^1,39,40^. The regional overburdened topsoil contains a large percentage of lateritic clay soil, which usually decreases with depth^39^. The vulnerabilities of the aquifers underlying the study area to the surface contaminants appear to be varied with different geological locations based on the morphologies of the subsurface^1,17,37^. That is, the subsurface geology affects the dispositional surface contaminants’ distribution and movement. However, the dumpsite and its environs do not have any protruding geological or terrestrial formations. The composition, texture, and colour of the topsoil vary greatly; in most areas, the soil is primarily composed of laterite and quartz grains, giving it a deep brown or reddish-brown colour.

### The climate of the study area

Understanding the interaction between climate change, air pollution, and profound impacts on human health, especially in the African continent, which is known for its diverse ecosystems and rich biodiversity^41^. This is because the adverse effects of climate phenomenal on pollution are particularly of great concern. In the Köppen Climate Classification, Kaduna, Nigeria belongs to the Aw climate, which is a tropical dry and wet savanna climate^42,43^. This implies that the region is highly associated with climatic fluctuations, where dry and rainy dominate the annual experiences with high temperatures, alternating from 27.6 °C to 40.5 °C^44^. Kaduna experiences about 16.3 °C as low temperature annually and 38.5 °C as high temperature annually, which is typical of tropical regions. However, the minimum and maximum annual temperature alternate between 24.1 °C and 40.5 °C in 2023^45^. However, there is a noticeable oscillation in the humidity levels, occurring at 84% rate maximally and 19% rate minimally^43^. Kaduna has constantly experienced relatively regular rainfall annually with average monthly precipitation ranging from a low of 0 mm (0”) to a high of 149 mm (5.87”)^45^.

Interestingly, there are dry spells in the area during certain months, with 24.9 days being the highest frequency of rainy days ever recorded during certain of those months. According to a recent study, the months with the coolest climate are December and January, with a mean monthly temperature of around 220 C, and the months with the hottest climate are March and April, with a mean monthly temperature of about 310 C^46^. The average annual rainfall in wet years is approximately 1800 mm, whereas in dry years it may be as low as 300 mm. The months with the highest rainfall are still July and August^45^. An average of 1000 mm of rain falls in the research region each year^45^. The rainy season usually extends from late April until October. The dry season usually starts in late October and ends in late April of the following year. This data sheds light on the behaviour of LLPs and the climate conditions in the area because the variations in precipitation may erode landfill covers, which could exacerbate sinkholes and pollutant migration^1^. Concerns also include geotechnical problems such as elevated infiltration rates, excessive pore-water pressure, soil liquefaction, and harm to soil covers and landfill liners^47^. Rain, spontaneous combustion, or excessive buildup can turn landfills into unstable terrain, increasing the risk of landslides or collapses3. Methane emissions from landfills are a contributing factor to climate change, while efforts are being made to turn these emissions into energy^48^. Severe weather conditions have the potential to interfere with garbage collection systems and contaminate the environment. The seasonal variability in climatic conditions, such as temperature fluctuations and rainfall patterns can significantly influence the generation and movement of leachate within landfills, ultimately affecting groundwater quality, the environment, and public health.

## Data acquisition and processing

The data acquisition was obtained in three different seasons with ultimate care monitoring the variation in the landfill subsurface. The first, second, and third data were acquired in mid-February with zero rain, mid-April with low rain, and mid-August with peak rain respectively.

### Materials

The materials used and the parameters used for field data acquisition include the earth’s resistivity and its components, magnetic compass, Global Positioning System (GPS), measuring tape, cutlass, leachate sample (LS), atomic absorption spectrometry (AAS), differential pulse anodic stripping voltammetry (DPASV), range poles, beakers, Flask, shovel and hoes, clean plastic bottles (0.75-litre), computer, software, etc.

### Physiochemical data

**Fig. 2:**
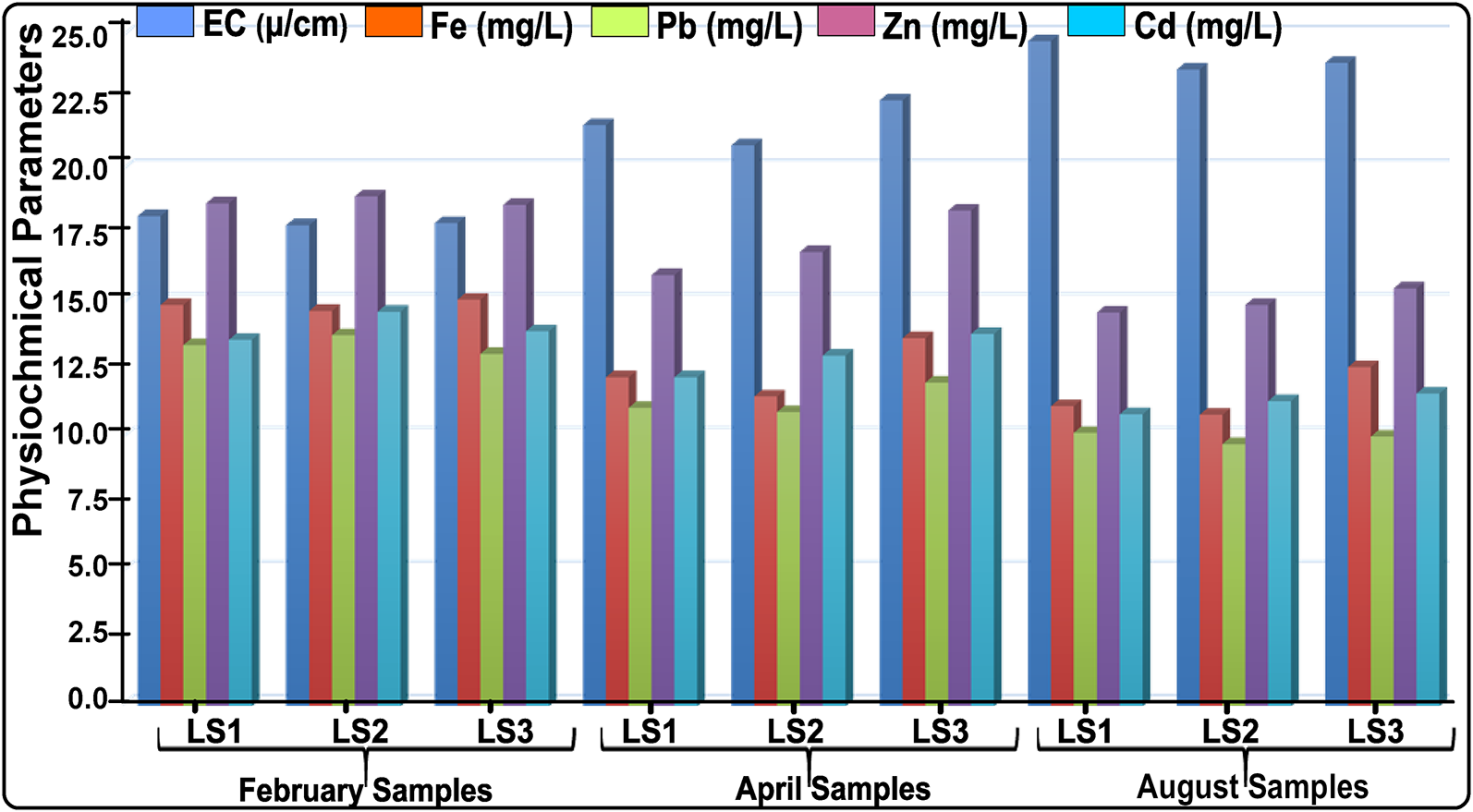
The Level of HMs and EC compositions present in the Investigated Landfill Leachaate Plume.

The physiochemical data of the LLPes involves three main stages, which include leachate sample (LS) collection, storage, and processing by Atomic Absorption Spectrophotometer (AAS). The leachate samples were collected in three different seasons and locations within the landfill at the same spot. Proper safety protocols such as the use of hand grooves and nose masks were observed during the LSs collection to prevent exposure to dangerous substances. The LSs were stored in appropriate containers (50 Cl plastic bottles) at the required temperature in the refrigerator for preservation. This is done to prevent microbial growth and chemical degradation to ensure the integrity of the LSs is maintained until analysis. The stored LSs were transferred into a plastic reagent bottle for AAS analysis. Then, the LSs were analyzed for various physicochemical properties such as electrical conductivity (EC) and heavy metals (HMs) concentrations. The HMs analyzed include zinc (Zn), lead (Pb), iron (Fe), and cadmium (Cd) using AAS. Thereafter, data interpretations were made alongside the seasonal comparison and their implications. This systematic approach was quite essential and challenging because the LSs obtained from the landfill were dirty and dangerous. The exact standardization and determination procedures for the different metals were followed and the results, analysis and graphical demonstration are presented in Table 1, Sect. 3.1, and Fig. 2, respectively.

### Geo-electrical resistivity

The data acquired using the ERT technique was quite challenging due to the movement of equipment within the landfill, especially during the rainy season. Electrical resistivity is a vital geophysical tool in which an electrical current is injected into the ground through steel electrodes in an attempt to measure the electrical properties of the subsurface^49,50^. Its technique is based on the response of the subsurface properties to the current flow, which operates on the principle of Ohm’s law as expressed in Eq. (1)

1 $$\:\:\:\:\:\:\:\:\:\:\:\:\:\:\varDelta\:V=IR$$

The resistivity meter used directly measures the response of subsurface materials, which was changed into the “electrical apparent resistivity ($\:{\rho\:}_{a}$), which can be expressed by Eq. (2)^50^.

2 $$\:{\rho\:}_{a}=K\frac{\varDelta\:V}{I}=RK$$

Where K is a geometry factor and it depends on the configuration of the four electrodes. However, the natural seasonal fluctuations usually influence the electrical conductivity of the soil due to the effect of soil water content^1^. The electrical study showed that the greater depth penetration required a wide electrode spacing, which implies that the value of the apparent resistivity is electrode-spaced-dependent. For Wenner’s alpha configuration, the K-factor can be defined by Eq. (3).

3 $$\:K=2\pi\:{\left[\left(\frac{1}{a}-\frac{1}{2a}\right)-\left(\frac{1}{2a}-\frac{1}{a}\right)\right]}^{-1}$$

Where, ($\:{r}_{AC}={r}_{BD}=a\:\:\text{a}\text{n}\text{d}\:\:\:{r}_{CB}={r}_{AD}=2a$), So that Eq. (3) can be transformed into (4) as:

4 $$\:\:\:\:\:\:\:\:\:\:\:\:\:\:\:\:\:\:\:K=2a\pi$$

Equation (4) is used to calculate the geometric factor (K-factor) for the Wenner alpha configuration. Following the data acquisition with 2D ERT of the Wenner-alpha array, the geophysical field data, was then fed into computer software (RES2DIV (version 3.6)) for analysis^51^. The root mean square (RMS) error is used to express the model block and fit correctness varies between 3.3% $\:-$ and 7.1%. Estimation of the exact depth of leachate plume occurrence is quite important because is one of the prime targets in geophysical exploration^52^, therefore, 3.0 m electrode spacing was used for adequate mapping of the LLP occurrence.

### On-site surveys and excavations

An on-site survey was conducted alongside soil excavations within and off the landfill. During the physical survey, some soils were excavated (drilled) to a depth of 1.5 m within and off the landfill for a critical analysis. This will provide insights into the interpretation of the geophysical and geochemical analysis for an adequate understanding of the possible reasons for the electrical conductive contrasts.

## Results presentations

### Presentations of the physiochemical results

**Fig. 3:**
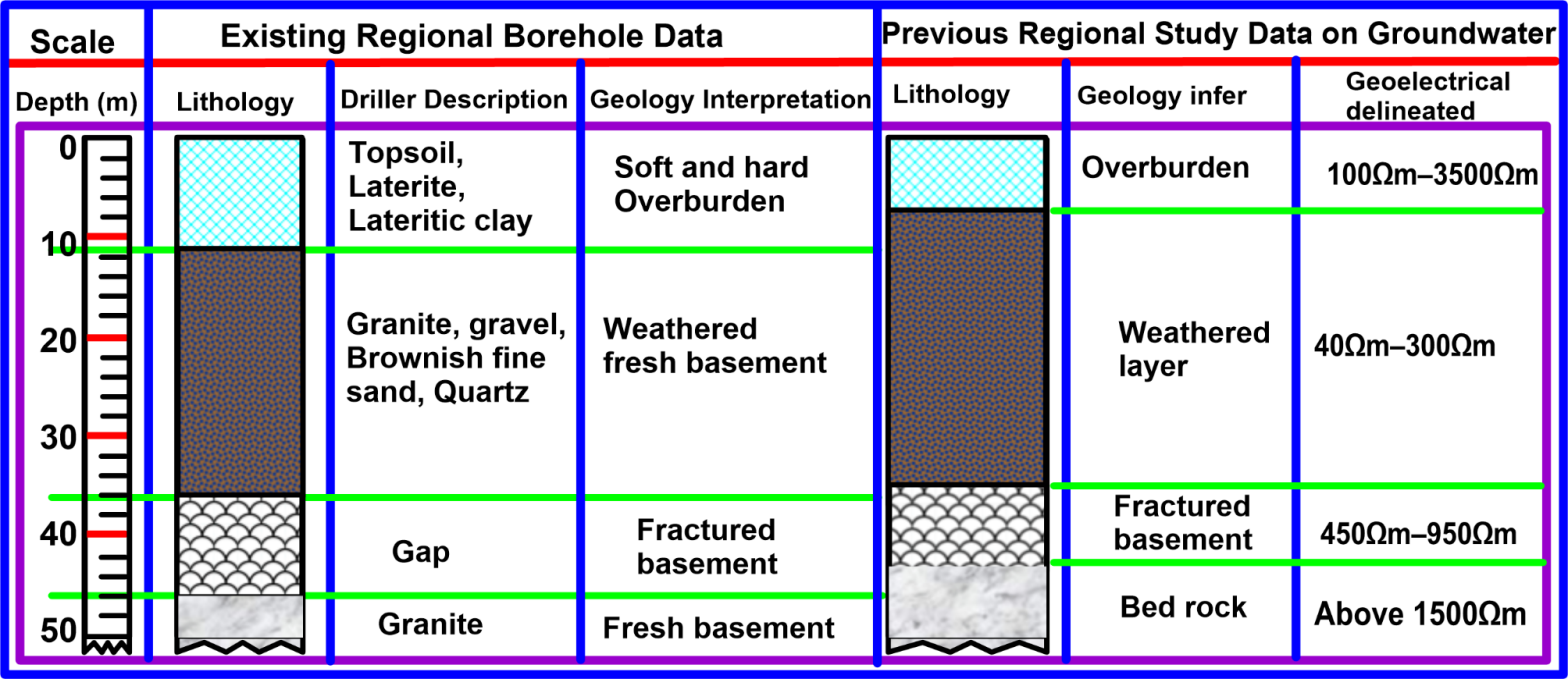
Geological well-logs of the Region. (courtesy of NWRI 1&2)^53^ and Compiled Regional Studies^30,39^.

**Fig. 4:**
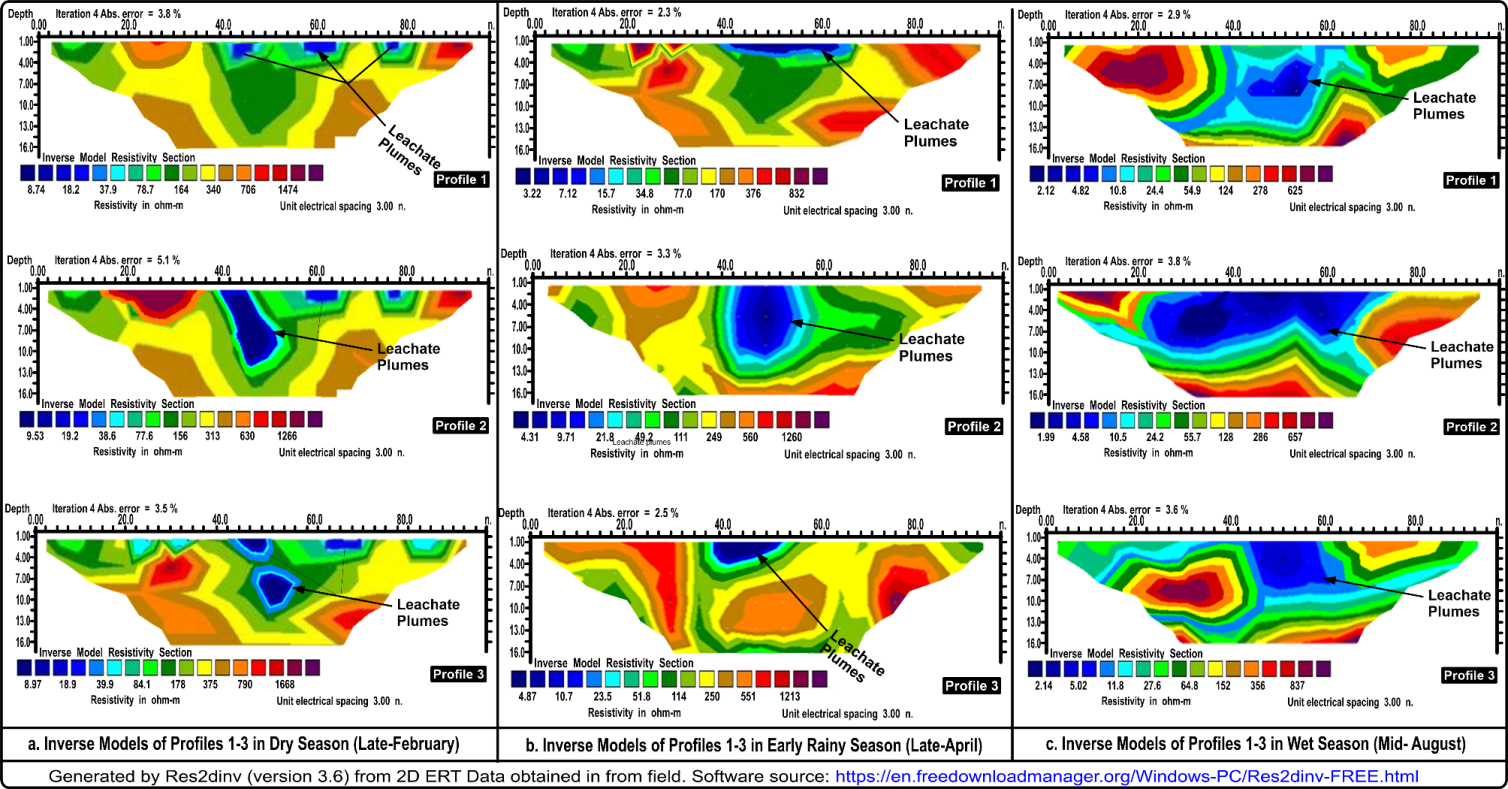
2D ERT Inverse Pseudo-section Models of Profiles 1–3 Acquired in three different Seasons, Namely dry, early rainy, and wet Seasons.

Table 1; Figs. 3 and 4 present the findings of the physicochemical analyses performed on the sample LLPs that were collected in three distinct seasons (February, April, and August). The findings show that the electrical conductivities (EC) and heavy metals (HMs) including Fe, Pb, Zn, and Cd are at incredibly high concentrations. Total dissolved solids (TDS), biological oxygen demand (BOD), and chemical oxygen demand (COD) are among the other physical factors that were looked at. The physiochemical parameters exhibit significant seasonal variation, as Table 1 illustrates. Seasonal variations in the values of physiochemical parameters occur between 24.87 ± 3.31 mg/L – 17.99 ± 1.92 mg/L for EC, while the investigated four HMs vary between 15.21 ± 3.12 mg/L – 10.91 ± 3.22 mg/L for Fe, 13.88 ± 2.77 mg/L – 9.81 ± 2.15 mg/L for Pb, 19.07 ± 3.68 mg/L – 14.73 ± 2.89 mg/L for Zn and 14.76 ± 1.37 mg/L – 10.93 ± 2.83 mg/L for Cd (Table 1). Figure 2 was created to provide a visual representation and graphical demonstration of how the few chosen HMs vary with seasonal variation, and highlight the trends in the variations, which inversely affect the groundwater systems. Figure 3 also displayed the seasonal fluctuations in the EC characteristic and the variations’ trends. These statistics and tables provide vital information on the state of open landfills, which harms soil, air quality, groundwater systems, and public health systems. The EC and HMs were selected to assess the influence of the landfill’s physiochemical properties alongside the geo-electrical resistivity survey to evaluate the subsurface electrical properties of the site. This will provide the overall impacts of the LLP contaminants on public health and general environmental organs such as groundwater systems, air condition and soil quality.

**Table 1:** The Seasonal Physiochemical Parameter and Characteristic of the Investigated Landfill leachate plume. Note: LS represents the leachate sample and 1, 2, &3 represent different seasons.

Parameter (Unit)	EC (ms/cm)	Fe (mg/l)	Pb (mg/l)	Zn (mg/l)	Cd (mg/l)
**Data Collection During Peak Dry Season (Mid-February)**					
LS 1	18.33 ± 2.19	15.01 ± 2.18	13.51 ± 3.71	18.81 ± 2.51	13.77 ± 2.01
LS 2	17.99 ± 1.92	14.79 ± 3.17	13.88 ± 2.77	19.07 ± 3.68	14.76 ± 1.37
LS 3	18.07 ± 2.32	15.21 ± 3.12	13.18 ± 3.09	18.75 ± 3.37	14.03 ± 2.18
**Data Collection During Pre-rany Season (Late-April**)					
LS 1	21.73 ± 2.12	12.31 ± 1.80	11.17 ± 1.81	16.13 ± 3.11	12.31 ± 1.80
LS 2	20.98 ± 3.32	11.59 ± 1.77	11.01 ± 1.33.	16.99 ± 3.78	13.12 ± 3.38
LS 3	22.66 ± 2.21	13.77 ± 2.11	12.11 ± 2.22	18.54 ± 3.43	13.93 ± 3.11
**Data Collection During Peak Wet Season (Mid-August)**					
LS 1	24.87 ± 3.31	11.23 ± 1.98	10.23 ± 2.38	14.73 ± 2.89	10.93 ± 2.83
LS 2	23.81 ± 2.42	10.91 ± 3.22	9.81 ± 2.15	15.01 ± 3.12	11.42 ± 2.56
LS 3	24.06 ± 2.27	12.69 ± 2.55	10.09 ± 2.22	15.63 ± 3.31	11.77 ± 4.18

### Presentations of the geo-electrical resistivity results

Figure 4 is a pictorial pseudo-section of 2D ERI resistivity inverse models with uneven distribution of subsurface resistivity values. The study area is underlain with different geological layers, starting with the topsoil (first) layer, laterite/dry clay (second) layer, weathered (third) layer, and fractured/bedrock basement (fourth/last) layer, which are highly conductive and resistive as the case may be with different resistivity values. A high pocket of electrical resistivity values varying between 42 Ωm – 1668 Ωm, 15.0 Ωm – 1066 Ωm, and 15.0 Ωm – 657 Ωm during dry, relative average, and wet seasons, respectively were encountered at the overburdened topsoil of the landfill ambient known as off-landfill, which was used as a controlled station. The fractured/basement rocks were found to be highly resistive, especially the bedrock with an infinite depth within and off the landfill. Within the landfill, a low pocket of electrical resistivity varying between 8.0 Ωm – 19.0 Ωm, 3.0 Ωm – 9.0 Ωm, and 1.5 Ωm – 5.0 Ωm during dry, relative average, and wet seasons, respectively, were encountered (Fig. 4). The delineated low subsurface resistivity was mapped as the LLP accumulated zones, occurring at a depth of 0.0 –12.0 m (Table 2). The geo-electrical results of the study also revealed the geoelectric layers comprising four to five distinctive geological, which include the topsoil layer, silty/clay/lateritic layer, weathered layer, fractured basement and fresh basement. The shallow low electrical resistivity contrasts aligned with the solid waste-hipped landfill and is linked to SW-oriented infiltration. In addition, the regional geological well-logs (borehole) data was used to support the study interpretation alongside the previous studies (Fig. 3). The information obtained from the regional borehole data and the extended survey profiles were also used as controlled stations for this investigation. Based on the subsurface ER values and morphologies in various geological locations of the study area, the surface contaminants due to LLPs appear to have significantly impacted the permeability of subsurface geoelectrical materials deriving from parent rock, which may also have been subjected to the varied conditions of weathering activities of the study area.

**Table 2:** Seasonal Variation in the geoelectrical parameters across all the profiles showing the horizontal distance and depth of occurrence of suspected landfill leachate plume contaminants.

Seasons	Off-landfill extension served as a Controlled station	Within the Landfill as Expression of the Degree of Impacts		
	ERV (Ωm)	ERV (Ωm)	Locations (m)	Depth (m)
	**Profile 1**			
Mid-February	42.0–1474	8.0–18.0	42.0–48.0	0.0–2.5
Mid-April	27.0–1066	3.0–9.0	40.0–60.0	0.0–2.5
MidAugust	15.0–625	2.0–5.0	42.0–58.0	0.0–10.0
	**Profile 2**			
Mid-February	43.0–1266	9.0–19.0	40.0–46.0	0.0–12.0
Mid-April	15.0–676	4.0–7.0	45.0–53.0	0.0–11.0
MidAugust	14.0–657	1.5–5.0	32.0–68.0	0.0–8.5
	**Profile 3**			
Mid-February	44.0–1668	8.0–19.0	43.0–51.0	0.0–11.5
Mid-April	16.0–726	4.0–10.0	38.0–50.0	0.0–4.0
MidAugust	15.0–437	2.0.0–6.0	45.0–60.0	0.0–9.0

## Discussion

From the overall results of the physiochemical and electrical resistivity investigations. The ERT in LLP detection provides a valuable technique for assessing contamination levels from waste dumps alongside the landfill physiochemical properties (LPPs), and how they vary in various seasons. The parameters of the HMs evaluated for leachate characterization include Fe, Pb, Zn, and Cd. The concentrations of spread of HMs present using ERT and LLPs techniques to measure the resistivity distribution and heavy metals as they migrate into the soil down to groundwater systems.

In the electrical resistivity (ER) technique, the subsurface features of the site delineated by the ER method include subsurface layers, conductive near-surface zones mapped as leachate plume accumulated, resistive shallow and deep zones delineated as lateritic topsoil and bedrock in some cases, and dipping linear conductor zones mapped as fracture zones just before the crystalline basement (Fig. 4). The lateritic topsoil mapped as conductive near-surface zones usually occurs off-landfill, while those with the landfill mixed with leachate plumes threaten its resistive nature. The shallower electrical conductivity delineated the boundaries of the waste disposal zone having low resistivity contrast varying between 1.5 Ωm – 19.0 Ωm across all the seasons with depths of 0.0–12.0 m, which established conductive electrical resistivity anomalies. The resistivity anomalies were found to be occurring minimally at the lowest electrical resistivity values within 1.5–5.0 Ωm, particularly during the heaviest August rainy season. The low ER values observed within the landfills were marked as leachate plume accumulated zones suggesting the presence of HMs within LLPs, which significantly influenced the electrical current flowing through the ground.

The investigation also shows that the LLPs have seeped down the ground beyond 10 m, which implies that the regional aquifer is potentially vulnerable to LLPs. This has placed the regional groundwater and the consumers on a pedestal of insecurity and high health risks, respectively. However, there are some intrusions within the landfill subsoil where ER values were occurring maximally within the ranges of 150 Ωm – 1260 Ωm, which may be due to regional intrusion rock (Fig. 4).

The extended survey (off-landfill) shows a high pocket of ER values varying between 15 Ωm − 1668 Ωm due to the non-existence of the LLPs, which is aligned with the previous studies carried out in a non-landfill site^38–40,54^. The ER values were observed to increase outwardly from 15 Ωm to 1668 Ωm confirming the horizontal impacts of the LLPs. The lower ER values were delineated mostly within the region of overburdened topsoil, which has an average thickness of 8.0 m. According to some remarkable studies, the integrity of groundwater is presumed to be threatened whenever the layers overlying the groundwater systems are compromised, posing a high risk to human health^1,7,26^. In addition, the regional borehole data indicates that the regional overburdened topsoil is usually characterized by hard lateritic, clay, and sandy soil^39,53^, which extends from 3.0 m to 10.0 m. With the low ER values encountered within landfills, the LLPs confirmed a significant influence of the LLPs due to the presence of HMs. Furthermore, the results of the excavations of soil depict soft and hard subsoil within and outside the landfill, respectively, which verified the results of the ER method. The drilling provides support information for an adequate understanding of the possible reasons for the minimal and maximal electrical conductive values present within and off the landfill. Also, high ER distributions across the site were noted to be varied across the seasons with the dry season having the highest ER values and the wet season with the lowest ER values.

In the physiochemical analysis technique, the leachate samples were analyzed with high, average, and low concentrations of HMs with clear seasonal trends in important parameters such as EC, Fe, Pb, Zn, and Cd. High concentrations of HMs are observed throughout the dry season, namely in February, and these concentrations drop with the onset of the wet season. In February, there were no rain events, and by April, there were early rainy periods. The maximal rain events that typically occur in August were seen to be decreasing continuously. High HM values were seen in the early rainy season, namely in February when there was little or no rainfall. These concentrations fall in April when there is relatively less rainfall, and fall even more in August when there is maximal rainy fall. The high concentration of HMs in the LLPs were indeed vary with the seasons as shown in Table 2, with the Fe decreases from 15.21 ± 3.12 mg/L to 10.91 ± 3.22 mg/L, Pb decreases from 13.88 ± 2.77 mg/L to 9.81 ± 2.15 mg/L, Zn decrease from 19.07 ± 3.68 mg/L to 14.73 ± 2.89 mg/L, and the Cd decreases from 14.76 ± 1.37 mg/L to 10.93 ± 2.83 mg/L, while the EC distractically increased from 17.99 ± 1.92 mg/L to 24.87 ± 3.31 mg/L.

These decrements and increments encountered in the LPPs are attributed to excess rainfall, which readily diluted the concentrations of HMs. This dilution effect can significantly lead to lower concentrations of HMs in the leachate and surrounding soil during wet seasons^55^. This is because the rainfall can wash away dust particles containing HMs transported within the landfill. However, parameters like EC gradually increase in content within the landfill due to the conductive nature of water, which is readily present in large volumes during the rainy season. The lower EC during this time may be due to fewer rainstorms, leachate volumes, and suspended solids content, which could have reduced the threat of groundwater pollution and associated health problems. The primary characteristic of the waste deposits that stands out from the chemical investigation is the high concentration of HMs, which are usually deposits substantially beyond the legal thresholds in Nigeria^10,11^. In particular, the descendant concentration trends of the three seasons differ despite holding identical HMs. The order of decrement in the HMs within the landfill is as follows EC > Fe > Zn > Pb > Cd, respectively. This most likely happens because the acid that was attacking was different^56,57^. In addition, the results of the chemical analysis show a good match with the ER results, which demonstrate seasonal variations in the distributions of HM concentration and ER values.

In addition, the changes in ER values potentially correlated with variations in HM concentration in LLP. Higher ER values indicate lower moisture content, while lower ER values suggest higher moisture content, which may affect the distribution and mobility of HMs. These enhance the flow of electrical current and the leaching of HMS into the ground soil down the groundwater systems. Therefore, understanding the impacts of HM distribution across the overburdened topsoil state is crucial for assessing the soil and groundwater contamination, which inversely triggers the potential health and environmental risks. The leachate contamination from the investigated non-sanitary landfills has significantly affected the integrity of the regional groundwater quality, which potentially threatens the public health of the nearby communities and the impacted groundwater consumers. In summary, the concentrations of the HMs within the investigated landfills decrease during the rainy season due to a high volume of dilution and transport effects. It is therefore essential to monitor and manage these pollutants to protect human and the environment’s health. The implications of seasonal variations on landfill leachate plumes and inversely on groundwater systems are significant to evaluate public health. Though the HMs presence in the leachate samples decreases with a wet season and increases with the dry season, the impact of these variations in LLPs seems to be more impactful during the wet season because of the movement of LLPs during this time. However, the impacts of high concentrations of HMs in the LLPs seem to be less impactful due to their limited movement during this period. The movement of contaminants present in leachate within the surface and subsurface can contaminate groundwater resources, leading to potential pollution of the surface and groundwater, which can damage the drinking water sources and ecological systems. The results of the physiochemical leachate analysis and geo-electrical resistivity monitoring underscore the importance of understanding how seasonal variations influence and control the LPP dynamics and the associated risks to groundwater systems and public health. The combined methods comprehensively and sufficiently identified and mapped the high-risk zones within the landfill, where leachate concentrations are highly elevated with well-migration pathways.

## Conclusion and recommendation

The geo-electrical resistivity and physiochemical water analysis coupled with the onsite survey and excavations for monitoring the LLPs in response to seasonal variations have provided valuable insights into the behaviour of leachate plumes within landfills and the implications on the public health and environmental vital organs such as groundwater systems. The study has successfully demonstrated the nature of the leachate plume dynamic with seasonal variations, which significantly influence the generation, distribution, and migration pathways of the LLPs. The tracks of trends of variations in the landfill subsurface electrical resistivity and the LLP distributions across the overburdened topsoil layer varying between 0.0 and 12.0 m. the combined techniques have successfully delineated the LLP accumulated zones, identifying the high-risk areas within the landfill, which were verified by on-site surveys and excavations. The subsurface resistivity value dispersed throughout the regional stratiform of the dumpsites has been greatly impacted by the presence of HMs and the geo-electrical survey data show a strong correlation with geochemical water analysis. However, it was quite challenging to move survey materials during the wet season. The delineated and analyzed subsurface electrical resistivity and HMs, respectively, vary between 1.5 Ωm – 19.0 Ωm, 9.81 ± 2.15 mg/L – 19.07 ± 3.68 mg/L, while the electrical conductivity varies between 17.99 ± 1.92 mg/L to 24.87 ± 3.31 mg/L. The high contrast in electrical properties between HMs and the host media provides the combined method to detect variations in subsurface resistivity and HMs. The excessive rainfall during the wet season, which occurs maximally around August exacerbated LLPs productions, emphasizing the need for prompt action to adequately manage waste and prevent water disruption in the region. In conclusion, the electrical resistivity and physiochemical leachate sample analysis techniques coupled with the on-site survey and excavation are suitable for imaging contaminated zones in various seasons, characterizing landfill geometry, and defining crystalline basement aquifer structures, assessing groundwater pollution risk, public health risk, and monitoring contamination behaviour. The findings underscored the need for proactive monitoring and remediation strategies to mitigate the potential risks posed by landfills to groundwater systems.

## Supplementary Information

Supplementary Information

## Data Availability

Data Availability Statement: The datasets used or analyzed during the current study are available from the corresponding author upon reasonable request.
