# Supplementary material for: Geoelectrical resistivity and geochemistry monitoring of landfill leachates due to the seasonal variations and the implications on groundwater systems and public health
Source: Sci Rep. 2024 Nov 3;14:26542. doi: 10.1038/s41598-024-77727-6 (PMC11532413; doi:10.1038/s41598-024-77727-6)
Supplement: Supplementary file 1 — Supplementary Information [file 41598_2024_77727_MOESM1_ESM.docx]

Dear Editor,

I am writing to you to clarify some issues regarding my Manuscript ID: 160ecbcf-cb8e-4edd-bb3a-7585af771302 v2.2. Concerning the message I received from you about the clarification on how the map in the figure was generated. I have explained it in the main manuscript, citing the sources of the software used in the references number 31, 32 and 51 (see the main manuscript), yet, I am still receiving the same message from you. I am afraid, maybe I do not understand what you want me to do. For instance, Fig 1 in the main manuscript was generated by the corresponding author from Google Earth using geographical coordinates acquired by a GPS and the picture of the landfill and was prepared using drawing tools like 22 (Version: 23.4 (x86)). Fig i-3 in this document was combined to produce Fig 1 in the main manuscript. All the maps in the figure were generated by me using the following sources;

1. Google Earth (Pro 7.1.2.2041 with Activator + Patch – KaRanPC. Google Earth): <https://www.google.com/earth/about/versions/>
2. Res2dinv (version 3.6): <https://en.freedownloadmanager.org/Windows-PC/Res2dinv-FREE.html>
3. Surfer 22 (Version: 23.4 (x86)): <https://surfer.software.informer.com/download/>

Thank you very much.

J. O. Alao (Corresponding author)

(alaojosephomeiza@gmail.com)

(+23408032734663)

Fig 1: Obtained from Microsoft Encarta Premium DVD 2009

Fig 2: Obtained from Kaduna State Geological Survey, Nigeria.

Fig 3: Photograph of the investigated Landfill
